# Assessment of Physical and Mechanical Parameters of Spun-Bond Nonwoven Fabric

**DOI:** 10.3390/polym16202920

**Published:** 2024-10-17

**Authors:** Inga Lasenko, Jaymin Vrajlal Sanchaniya, Sai Pavan Kanukuntla, Arta Viluma-Gudmona, Sandra Vasilevska, Sanjay Rajni Vejanand

**Affiliations:** 1 Institute of Mechanical and Biomedical Engineering, Faculty of Civil and Mechanical Engineering, Riga Technical University, 6B Kipsala Street, LV-1048 Riga, Latvia; 2 Faculty of Economics and Social Development, Latvia University of Life Sciences and Technologies, 2 Liela Street, LV-3001 Jelgava, Latvia; sandra.vasilevsky@gmail.com

**Keywords:** spun-bond, nonwoven, FPP2 mask, physical and mechanical properties, breathability, filtration

## Abstract

The selection of an appropriate fabric for technical applications, such as protective masks, hinges on a thorough understanding of the fabric’s physical and mechanical properties. This study addresses the challenge of selecting the optimal material structure for the upper layer of a protective mask, aiming to ensure adequate breathability while providing effective filtration against airborne particles and contaminants. We assessed and compared the physical–mechanical properties of five polymer spun-bond nonwoven fabrics from different suppliers. Our comprehensive evaluation included, as follows: a visual inspection; light permeability analysis; mass and thickness measurements; elongation and tensile strength tests; breathing resistance assessments; and filter penetration tests with paraffin oil. The results revealed significant variations in performance among the samples, with one fabric consistently outperforming the others across multiple parameters. Notably, this top-performing fabric met or exceeded the EN 149:2001+A1:2009 standard for breathing resistance and filtration efficiency and, in combination with additional filter layers, met the requirements or exceeded class FFP2 (filtering face piece). This study underscores the importance of meticulous material selection and quality control in optimizing PPE (personal protective equipment) performance and user safety, providing valuable insights for mask manufacturers and healthcare professionals.

## 1. Introduction

Nonwoven fabrics have emerged as essential components in various applications, including personal protective equipment like face masks [1]. Composed primarily of polymers, these fabrics offer a unique combination of desirable properties such as durability, filtration efficiency, microbial barrier function, liquid repellence, and softness [2,3]. These fabrics find applications in a wide range of fields, including embankments and composite materials [4]. Unlike knitted and woven fabrics, nonwoven fabrics possess a unique microstructure [5,6], characterized by randomly oriented fibers, which significantly influences their mechanical behavior [7,8]. Among the various nonwoven fabric manufacturing techniques, spun-bond technology stands out due to its ability to produce lightweight yet robust materials.

The assessment of a fabric’s physical and mechanical parameters is crucial for selecting materials suitable for technical applications, particularly for producing personal protective equipment like face masks [9,10,11]. Filtering face piece (FPP2) masks, typically multilayered, rely heavily on their first layer for primary protection. This layer, often made from various 100% polymer materials and classified as a filter material, has garnered significant attention [12,13,14,15,16,17]. Face masks are essential in public settings to curb virus transmission, as they effectively reduce the distance and concentration of virus-laden particles expelled from infected individuals [18,19]. Masking of the face considerably decreases the distance traveled and concentration of virus-carrying particles breathed from the noses and mouths of infected persons [20,21,22].

The effectiveness of masks depends on several factors directly related to the physical and mechanical properties of the filtering fabrics. These include, as follows: the fabric’s structure, fiber diameter, and pore size distribution, which influence filtration efficiency; the fabric’s porosity and thickness, which affect breathability and user comfort; the fabric’s tensile strength and elongation properties, which determine the mask’s ability to maintain its shape and fit during use; and the material’s mechanical strength and resistance to degradation, which impact the mask’s durability and capacity to withstand repeated use and cleaning. Understanding these properties is paramount to ensuring mask effectiveness in real-world applications, where masks must balance filtration efficiency with user comfort and long-term performance.

The demand for personal protective equipment, including polymer filters, has surged in recent times. Research has demonstrated the efficacy of these polymer filter materials, with studies identifying materials capable of filtering over 95% of virus-containing particles [23,24]. However, while these studies focus on the overall filtration efficiency of materials in ideal settings, they often overlook the individual properties of each mask layer. This oversight is crucial, because the quality of each layer directly impacts the overall protective capabilities of the mask. Research conducted in the EU underscores the importance of evaluating the strength and structural uniformity of polymeric filter nonwoven materials used in masks [3,25]. For instance, light transmission through the filter fabric is a critical factor influencing the mask’s protection effectiveness, particularly for tiny particulates (about 150 nm) that can easily follow airflow in loosely woven fabrics [26,27]. This highlights the importance of considering ergonomic design in conjunction with material properties [28,29,30,31].

Despite the existing knowledge, a critical gap exists in understanding the relationship between the overall quality of filter fabrics and the specific physical and mechanical properties of individual polymer spun-bond nonwoven fabric layers. While manufacturers often emphasize surface density as a key indicator, fabrics with identical surface densities can exhibit varying degrees of light permeability, directly affecting breathability [32,33]. Therefore, a comprehensive evaluation of each layer’s physical performance, especially for spun-bond nonwoven fabrics (outer layer), is essential during the design phase of personal protective equipment (PPE) masks.

This study aims to assess and compare the physical and mechanical properties of various spun-bond nonwoven fabrics used in protective masks. By examining these properties, we seek to provide insights into how material characteristics influence mask performance, particularly in terms of filtration efficiency, breathability, and durability. This information is crucial for manufacturers in selecting appropriate materials and for users in understanding the protective capabilities of different mask types. To achieve this, we conducted a comprehensive assessment of five polymer spun-bond nonwoven fabrics, evaluating their visual characteristics, light permeability, mass, thickness, elongation, and tensile strength. A breathing resistance test, and filter penetration test with paraffin oil were also conducted.

The three objectives of this study are as follows: to comprehensively assess the physical and mechanical properties of five different spun-bond nonwoven fabrics used in the upper layer of protective masks; to evaluate their performance in terms of breathability and filtration efficiency when incorporated into multi-layered mask structures; and finally, to compare these results against FFP standards to determine their suitability for high-performance protective masks. By achieving these objectives, we aim to provide valuable insights into the optimal selection of materials for protective mask manufacturing, potentially improving both the effectiveness of personal protective equipment and user comfort.

## 2. Materials and Methods

### 2.1. Materials and Sample Preparation

Spun-bond nonwoven fabric (100% PP (polypropylene); 50 ± 2 g/m^2^; black color; width, 19.0 ± 0.3 cm) was purchased from the market. The following four types of specimens were bought and designated: **S1** (Polycorp LLC, Yudina Str, 11, Dnipro 4900, Ukraine); **S2** (UAB MERBONAS, Bičiũnog 39-1, LT47240, Kaunas, Lithuania); **S3** (LENA GLOBAL MEDIKAL, Ostim Osb Mah 1568 Cad No:43, 06374 Ankara, Turkey); and **S4** (Lentex, Spółka Akcyjna, 42-700 LUBLINIEC, ul. Powstańców Śląskich 54, Warszawa, Poland). Each had a length of 6 m. All nonwoven fabrics were recommended by suppliers for the manufacture of PPE, with a protection class of FFP2. In order to assure that all specimens passed the required tests, all studied parameters of the spun-bond nonwoven fabrics were compared to a reference-standard, spun-bond nonwoven fabric obtained from NV EVOLUTIA NONWOVENS (Carretera de Denia S/N, 03830, Muro de Alcoy (Alicante), Spain), referred to as **S5** throughout the rest of this article. This S5 layer (within the structure of the mask, class FFP2) passed the required tests and was certified as no. 22/5161/00/0161, 11 July 2022 (valid until 11 July2027). For each test described below, five samples of each fabric type (S1–S5) were tested to ensure statistical significance.

### 2.2. Visual Inspection and Microscopic Examination

Visual inspections (including a description of the samples (microscopic examination) and determination of the light permeability/propagation of nonwoven fabrics) were performed according to the standards EN14683:2019+AC:2019 [34] and EN 149:2001+A1:2009 [35] and were conducted on a Leica M165 C (GE, 16.5:1 zoom, 40× magnification) up to a 906 lp/mm resolution (with a 2.0× objective). Before testing, all specimens were kept under normal controlled conditions for 24 h (standard temperature, relative humidity, and pressure) according to ISO 139:1973 [36], as follows: a temperature of 25 ± 10 °C (21 °C); relative air humidity of 45–80% (W (% RM) = 60%); and an atmospheric pressure of 84.0–106.7 kPa (630–800 mm Hg; 760 mm Hg).

### 2.3. Light Permeability Analysis

Microscopic examination and determination of the light permeability/propagation of the nonwoven fabric specimens was performed on a Mitutoyo QI-A2010D (manual vision-measuring machine) (Mitutoyo, Wroclaw, Poland). The Mitutoyo QI-A2010D was connected to QIPAK software (V 5.0). The fabric specimens were kept on a table and light was passed through from the bottom, with a fabric image appearing in the camera. The whole image was captured using a stitching tool. To measure the light permeability, the captured images were imported into ImageJ software (open source), version 1.53e, 2021 (National Institutes of Health, Bethesda, MD, USA). First, the images were converted into 8-bit format (black and white). Using an enhancement tool, the images were enhanced by 0.5% to ensure a clear difference between white and black. A threshold tool was used to identify the white, as indicated by light passing through the fabric, and was visualized in a light histogram, which, when adjusted, clearly separated blocked light from light passing through the fabric. The diameter of the inspected fabric was 15.0 ± 0.1 cm.

### 2.4. Mass and Thickness Measurements

Mass determination was considered according to the mass per unit area (weight) of the fabric and the standard ASTM D3776-20 [37]. The machines used for mass determination were KERN PCB M laboratory scales (KERN & Sohn GmbH, D-72336, Balingen, Germany) with an accuracy class as follows: II high, maximum of 200 g, discreteness of 0.01 g; calibration certificate number M0805K24, 02 April 2024. A measuring tape was used for 0–3 m, with two scales, verification number G305V24, 2 April 2024.

The thickness of the nonwoven spun-bond fabric material was determined using a digital micrometer QuantuMike instrument, series 293-IP65 (Mitutoyo, Wroclaw, Poland) with a discreteness of 0.001 g; a personal computer calibration was conducted at the start according to ISO 9073-2:1995 [38].

### 2.5. Elongation and Tensile Strength Tests

The elongation and tensile strength were determined according to the standard ISO 9073-3 [39] strip method. The standard test method for breaking force and elongation of nonwoven materials, WSP 110.4, was implemented on Mecmesin’s Multi-Test 2.5-I with a specimen size of 5 × 10 cm, a speed of 100 mm/min, at 2.5 kN and 1–1000 mm/min (0.04–40 in/min) (PPT Group UK Ltd., t/a Mecmesin, Newton House, Spring Copse Business Park, Slinfold, West Sussex, RH13 0SZ, UK).

The tensile tests were conducted in two directions, the machine direction (MD) and cross-machine direction (CMD). These terms refer to the orientation of fibers in nonwoven fabrics resulting from the manufacturing process. The machine direction (MD) is parallel to the direction of fabric production and typically aligns with the predominant fiber orientation. The cross-machine direction (CMD) is perpendicular to the MD. Testing in both directions is important, as nonwoven fabrics often exhibit anisotropic mechanical properties due to preferential fiber alignment during manufacturing.

### 2.6. Breathing Resistance Assessment

**Figure 1:**
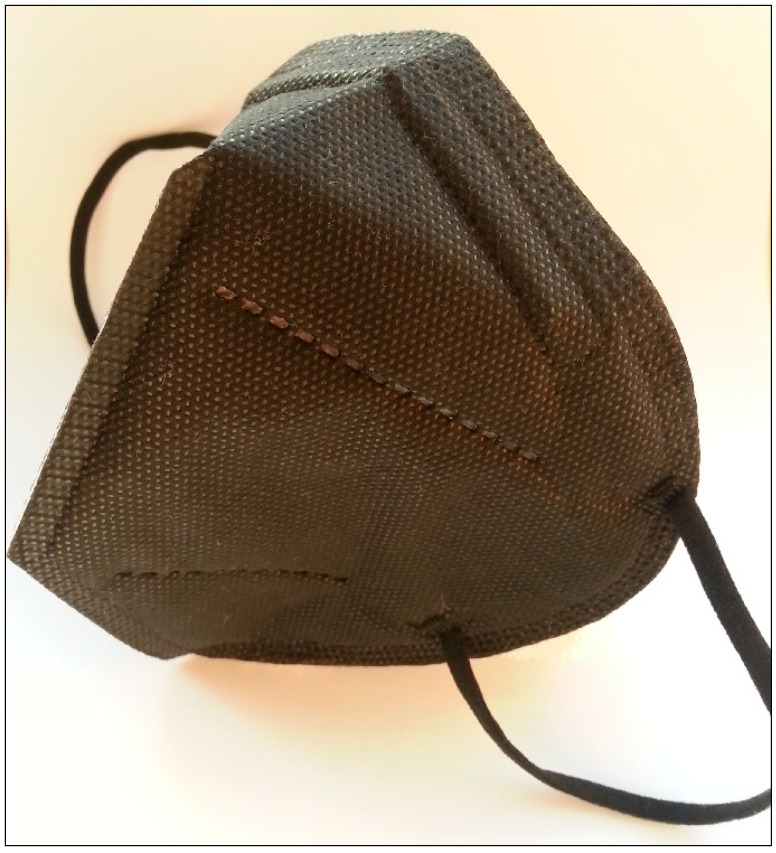
Image of the prepared multi-layered FFP2 respirator mask utilized in this study.

This study employed a multi-layered filter system, incorporating spun-bond nonwoven fabric as the upper layer (S1, S2, S3, S4, and S5). The complete filter assembly, comprising five layers, is protected under a registered patent application (LVP2023000040, 28 April2023) for a 3D multilayer protective product. The other layers in the mask were made of hot air cotton (45 ± 2.5 g/m^2^); melt-blown nonwoven fabric (25 ± 2 g/m^2^); melt-blown nonwoven fabric (25 ± 2 g/m^2^); and spun-bond nonwoven fabric (30 ± 2 g/m^2^). These textile materials were obtained from NV EVOLUTIA NONWOVENS (Carretera de Denia S/N, 03830, Muro de Alcoy (Alicante), Spain). The manufacturing technology and materials used in this study adhered to the production requirements for FFP2 masks. In total, 100 masks were manufactured. Figure 1 shows the prepared multi-layered mask.

Testing was conducted using the Dr. Wiesner Lab Test System for Respirator Masks (Weststraße 4, Remshalden, Germany) to evaluate the masks’ breathability and paraffin oil penetration. The breathability assessment followed the EN 149:2001+A1:2009 [35] standard, consisting of the following three primary tests:

- Average resistance to inhalation at 30 L/min;
- Average inhalation at 95 L/min;
- Average resistance to exhalation at 160 L/min.

For each test, measurements were taken from five different locations on the mask material. The airflow meter was calibrated to ensure measurement accuracy. The filter material was mounted on the test apparatus, creating an airtight seal. The required airflow was set in the appropriate direction (inhalation or exhalation), and the system was allowed to stabilize for one minute before measuring the pressure difference across the filter material. This process was repeated five times with fresh samples, and the average resistance was calculated from these measurements.

### 2.7. Filter Penetration Tests

The paraffin oil penetration test was performed by mounting the mask material on the testing apparatus and setting the airflow to 95 L/min. Paraffin oil aerosol was generated at a concentration of 20 mg/m^3^ ± 5 mg/m^3^ and allowed to flow through the filter for three minutes, in accordance with EN 149:2001+A1:2009 [35]. The concentration of paraffin oil was measured before and after the filter, and the penetration percentage was calculated. This test was repeated five times to ensure statistical validity.

The exposure to 120 mg of paraffin oil (clogging test) was conducted similarly to the paraffin oil penetration test. The paraffin oil aerosol generator was calibrated to deliver a total of 120 mg. With the airflow set to 95 L/min, the test continued until 120 mg of paraffin oil had been delivered to the filter. The maximum penetration percentage was then calculated. This process was also repeated five times to ensure statistical validity.

## 3. Results and Discussions

### 3.1. Visual Inspection and Microscopic Examination Results

**Figure 2:**
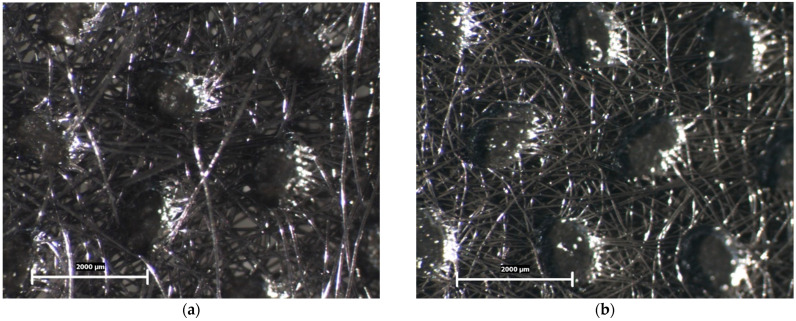
Microscopic surfaces of samples for morphological analysis: (**a**) S1; and (**b**) S5. Scale 2000 μm.

Spun-bond nonwoven fabric (S1, S2, S3, S4, and S5) can be morphologically described as a three-dimensional, hydraulically entangled, nonwoven composite structure composed of a nonwoven fibrous web comprising hydraulic entanglement. It is an embossed mat with precisely distributed areas of thermal fusion. Spots cover the whole mat plane and form a uniform pattern on its surface. According to the microscopic examination, the spun-bond nonwoven fabric tested in this study was characterized by an average spot size of 650 ± 10 × 1300 ± 20 μm, a distance between spots of 1750 ± 25 μm, and an average number of microfibers per unit square element on the mat’s surface (Figure 2a,b).

The microscopic examination of the spun-bond nonwoven fabric revealed crucial information about its structural characteristics, which directly influence its performance as a protective barrier in FPP2 masks. The three-dimensional hydraulically entangled structure provides a complex network of fibers that enhances the fabric’s filtration capabilities [1,40]. This structure is particularly important for trapping airborne particles and contaminants, which is a primary function of the outer layer of a protective mask.

The embossed mat, with distributed areas of thermal fusion, contributes to the fabric’s strength and durability. The uniform pattern formed by these fusion spots likely provides a balance between structural integrity and flexibility, which is essential for mask comfort and fit. The precise measurements of spot size and inter-spot distance indicate a consistent and controlled manufacturing process, crucial for maintaining uniform quality across the fabric. The presence of microfibers in the mat’s surface increases the surface area available for particle capture, potentially enhancing the fabric’s filtration efficiency.

**Figure 3:**
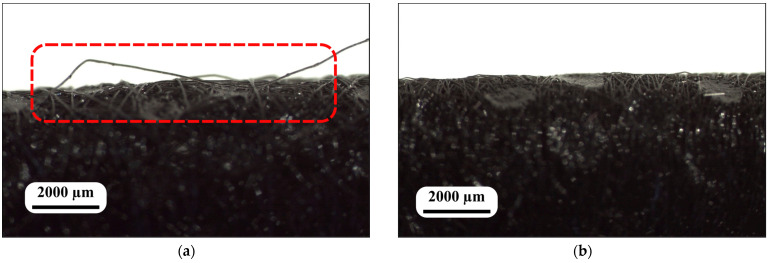
Microscopic lateral curved surface of samples: (**a**) S1 with dangling filament (red); and (**b**) S5.

The visual inspection results showed that the parts of the samples that come into contact with the user did not have burrs. In specimen S1, the surface of the spun-bond nonwoven fabric was characterized by the presence of single dangling filaments (red—Figure 3a). In specimen S5, the surface of the spun-bond nonwoven fabric was characterized by a 100% absence of single dangling filaments (Figure 3b).

The visual inspection results provide valuable insights into the surface characteristics of the spun-bond nonwoven fabrics, which are crucial for both user comfort and filtration efficiency. The absence of burrs in the parts of the samples that come into contact with the user is a positive finding, as burrs can cause skin irritation and discomfort, potentially leading to reduced compliance in mask wearing [27,41]. This smooth surface is essential for ensuring user comfort during prolonged use of the mask.

The presence of single dangling filaments in specimen S1 could potentially affect the fabric’s performance by creating inconsistencies in the fabric structure and compromising filtration efficiency. In contrast, specimen S5’s complete absence of dangling filaments suggests a higher level of manufacturing precision and quality control. This characteristic is likely to contribute to more consistent filtration performance, improved durability, and better user perception. The difference between S1 and S5 in this aspect highlights the importance of manufacturing processes and quality control in producing high-quality spun-bond nonwoven fabrics for use in protective masks.

The S2, S3, and S4 samples did not pass the visual inspection because they also had dangling filaments (area: 19.0 ± 0.3 cm (width) × 1 m (length), with about five dangling filaments, according to EN 149:2001+A1:2009 [35]).

### 3.2. Light Permeability Analysis

To conduct a detailed comparative analysis and study of the morphological structure of the spun-bond nonwoven fabrics, light permeability measurements were performed using the Mitutoyo QI-A2010D (2D color measuring system) described in Section 2. The results are presented in Table 1, showing the percentage of light passing through each fabric sample with a tolerance of ±5%.

The statistical analysis revealed no significant difference in light permeability among samples S1, S2, S3, and S4 (*p* > 0.05, one-way ANOVA). However, sample S5 showed significantly lower light permeability compared to the other samples (*p* < 0.001, one-way ANOVA followed by Tukey’s post hoc test).

The measurements reveal varying degrees of light permeability across the five samples. Sample S3 exhibited the highest light permeability at 15.80 ± 0.84%, followed closely by S4 at 15.05 ± 0.7%. Samples S1 and S2 showed similar values of 14.84 ± 0.7% and 14.44 ± 0.68%, respectively. Notably, sample S5 demonstrated the lowest light permeability at 10.09 ± 0.5%, which was significantly lower than that of the other four samples. This variation in light permeability suggests differences in the fabric structure and density among the samples, despite their similar specifications (100% PP (polypropylene); 50 ± 2 g/m^2^).

Lower light permeability, as observed in S5, suggests a denser fabric structure with potentially better filtration capabilities. This could translate to improved particle capture efficiency, which is important for the outer layer of an FPP2 mask. However, it is important to note that while lower light permeability might enhance filtration, it could also impact breathability. The higher light permeability observed in samples S1–S4 might indicate a more open structure, potentially offering better breathability but possibly not enough particle filtration efficiency (due to the 3D structure of spun-bond fabric). These results underscore the importance of balancing filtration and breathability in mask design and suggest that S5 might offer the best filtration properties among the tested samples.

**Figure 4:**
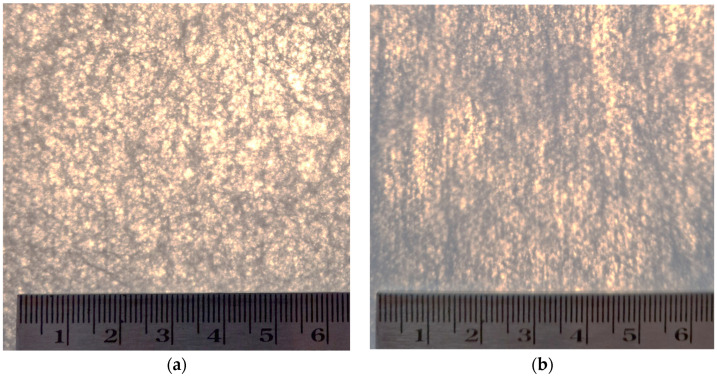
Visual comparison of light permeability/propagation of nonwoven fabrics: (**a**) S2; and (**b**) S5. Scale in cm.

For a detailed morphological comparison of the two-dimensional surfaces of the spun-bond nonwoven fabrics, the uniformity of color transmission (white/black) was considered. The nonwoven fabrics were placed on a white sheet of paper (under standard office illumination (400 lux)); the results are shown in Figure 4.

Figure 4 reveals distinct differences in the light permeability and uniformity of the two samples. Sample S2 (Figure 4a) exhibits a more varied pattern of light transmission, with noticeable light spots distributed across the fabric surface. These light spots appear as white or lighter areas against the overall darker background of the fabric. The distribution of these spots seems relatively uniform, but their presence indicates areas of higher light permeability within the fabric structure.

In contrast, sample S5 (Figure 4b) presents a more homogeneous appearance. The image shows a more consistent dark color across the fabric surface, with fewer and less pronounced light spots visible. This suggests that S5 has a more uniform structure with fewer areas of high light permeability compared to that of S2, despite the fact that the two samples have almost the same mass (as shown in Table 2). The overall darker appearance of S5 also indicates lower overall light transmission, which aligns with the quantitative light permeability measurements presented in the previous result.

These visual observations of light permeability provide valuable insights into the structural uniformity and potential filtration characteristics of the spun-bond nonwoven fabrics. The more varied light transmission pattern observed in S2 suggests a less uniform fabric structure, which could result in inconsistent filtration performance across the material. Areas of higher light permeability might correspond to regions where particles could more easily penetrate the fabric, potentially reducing its overall effectiveness as a protective barrier. The S1, S3, and S4 samples did not pass the visual inspection because they also did not have a homogeneous structure (according to EN 149:2001+A1:2009 [35]).

On the other hand, the more homogeneous appearance of S5 indicates a more consistent fabric structure. This uniformity is likely to contribute to more reliable filtration performance across the entire fabric surface. The lower overall light transmission of S5 also suggests a denser structure, which could enhance its particle capture efficiency. However, it is important to consider that while a denser structure may improve filtration, it could also impact the breathability. The balance between these factors is crucial in the design of effective protective masks.

### 3.3. Mass and Thickness Measurements

The differences in light permeability through the nonwoven fabrics were further investigated by determining the mass per unit area and thickness of the specimens. Table 2 presents the results of these measurements for all five samples (S1–S5).

The mass per unit area of the samples ranged from 48 ± 2 g/m² to 53 ± 2 g/m², with a tolerance of ±5%. Sample S1 showed the highest mass at 53 ± 2 g/m², followed by S3 at 51 ± 2 g/m². Samples S2 and S5 had similar masses of 50 ± 2 g/m² and 50 ± 2 g/m², respectively, while S4 had the lowest mass at 48 ± 2 g/m². These values are all within the expected range for the specified 50 ± 2 g/m² fabrics, with slight variations that could impact performance.

The thickness measurements, reported with a tolerance of ±10%, show more pronounced differences among the samples. S1 had the highest thickness at 0.29 ± 0.03 mm, significantly higher than the other samples. S2 and S3 had similar thicknesses of 0.25 ± 0.02 mm and 0.25 ± 0.02 mm, respectively. S4 was slightly thinner at 0.24 ± 0.02 mm, while S5 showed the lowest thickness at 0.21 ± 0.02 mm. This variation in thickness, despite similar masses, suggests differences in the fabric density and structure among the samples.

The mass and thickness measurements provide information about the structural characteristics of the spun-bond nonwoven fabrics, which can significantly influence their performance as protective materials. The variations in the mass per unit area, although relatively small, can affect the fabric’s filtration efficiency and breathability [42]. A higher mass, as seen in S1, might indicate a denser fabric structure, potentially offering better filtration but possibly at the cost of reduced breathability.

The thickness measurements reveal more substantial differences among the samples. The notably higher thickness of S1, combined with its higher mass, suggests a less compressed, possibly more porous structure. This could explain its higher light permeability observed in previous tests. Conversely, S5’s lower thickness, despite a similar mass to other samples, indicates a more compressed, denser structure. This aligns with its lower light permeability and potentially better filtration properties. These findings highlight the complex relationships among the fabric structure, mass, and thickness, and their potential impacts on the filtration efficiency and breathability in protective mask applications.

### 3.4. Elongation and Tensile Strength Results

**Figure 5:**
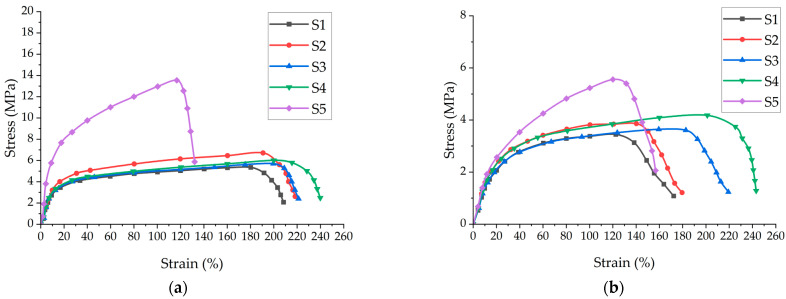
Representative force–elongation graph of samples S1 to S5 in: (**a**) MD; and (**b**) CMD.

Figure 5 presents the stress(σ)–strain(ε) curves for the nonwoven spun-bond fabric samples (S1–S5) in both the machine direction (MD) and the cross-machine direction (CMD). The graphs reveal a characteristic crimp zone, which is attributed to the initial alignment of nonwoven fibers under load. This crimp zone is primarily caused by the lower bending stiffness of the fibers relative to their elongation stiffness.

Table 3 provides the detailed tensile test results for all five specimens in both the MD and the CMD. In the MD, the ultimate tensile strength ranged from 5.4 ± 0.4 MPa (S1) to 13.5 ± 0.6 MPa (S5), with elongation percentages between 135 ± 7% (S5) and 239 ± 19% (S4). In the CMD, the ultimate tensile strength varied from 3.4 ± 0.3 MPa (S1) to 5.6 ± 0.3 MPa (S5), with elongation percentages ranging from 153 ± 8% (S5) to 241 ± 21% (S4). Notably, S5 demonstrated the highest tensile strength in both directions but the lowest elongation, while S4 showed the highest elongation in both directions.

For comparison, it is worth noting the industry standards for spun-bond nonwoven fabric (50 ± 2 g/m²) used in face masks, as specified by the Corporate Standard of TESTEX TS M003-2020 [43]. This standard stipulates minimum values for the ultimate tensile strength of ≥10 MPa in the machine direction (MD) and ≥5 MPa in the cross-machine direction (CMD). For elongation, the minimum values are set at ≥120% in the MD and ≥130% in the CMD. These reference values provide a benchmark for evaluating the performance of the tested samples in the context of face mask applications.

**Figure 6:**
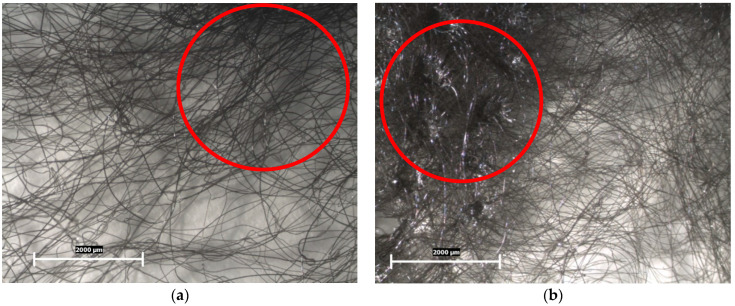
The structure of the nonwoven material at the point of rupture with density of fibers (red): (**a**) S1; and (**b**) S5.

The characteristic structure of the nonwoven material at the point of rupture is illustrated in Figure 6, comparing samples S1 and S5. Figure 6a, corresponding to sample S1, shows the material’s structure above the rupture point with a low density of fibers (marked with red). In contrast, a markedly different result can be seen above the rupture point of specimen S5 (with a high density of fibers, marked with red), as shown in Figure 6b. The S5 specimen was not completely torn into two halves, with pronounced partial parallelization of the filaments visible at the rupture locations.

The elongation measurements reveal that specimen S5 exhibited almost half the elongation of specimens S1, S2, S3, and S4 in both the MD and the CMD. This significant difference suggests that the filament fibers in S5’s structure experienced a more uniform load distribution, with elongation occurring similarly in both two-dimensional directions. This behavior indicates the absence of a pronounced 3D structure in S5, contrasting with the elongation patterns observed in specimens S1, S2, S3, and S4. These findings correlate well with the previously studied values of thickness and surface density of the nonwoven materials, highlighting the distinct characteristics of S5 compared to the other specimens.

Microscopic observations of the rupture process provide further insights into the material behavior. The “rupture” in these materials is not an instantaneous event but a process that occurs over time. During the observed period, approximately 40–60% of the stretched microfibers either failed or disconnected from the spots where they were originally melted together. This progressive failure mechanism contributes to the overall elongation and rupture characteristics of the nonwoven fabrics.

The stark contrast in rupture behavior between S1 and S5 provides valuable insights into their structural integrity and potential performance in mask applications. S5’s incomplete tear into two halves and the pronounced parallelization of partial filaments at the rupture points suggest a denser, more uniform, and potentially stronger fiber alignment. This characteristic could contribute to better filtration efficiency and structural integrity under stress, which are crucial for maintaining protective properties during mask use.

However, the significantly lower elongation of S5 compared to the other samples indicates less flexibility, which could affect mask fit and comfort. The uniform load distribution and two-dimensional elongation in S5 suggest a more planar structure, potentially offering consistent filtration across the fabric but possibly at the expense of conformability to facial contours. The progressive nature of the rupture process, with 40–60% of the microfibers failing over time, highlights the importance of considering not just the ultimate strength but also the fabric’s behavior under prolonged stress in mask design. These findings underscore the need to balance filtration efficiency, structural integrity, and flexibility in selecting optimal materials for protective mask applications.

### 3.5. Breathing Resistance Assessment

Table 4 presents the average resistance to inhalation for the five multi-layered mask samples (S1–S5), each incorporating a different spun-bond nonwoven fabric as the upper layer. The measurements were taken at two different airflow rates, 30 L/min and 95 L/min, which are crucial for assessing the breathability of the masks under different respiratory conditions. For the following inhalation and exhalation resistance measurements, it is important to note that the expanded uncertainty is ± 10% of the value of the measured result, for a probability of coverage of 95%. This uncertainty applies to all measurements reported in Table 4 and Table 5.

At the lower airflow rate of 30 L/min, the resistance values ranged from 0.61 ± 0.02 mbar (S5) to 0.81 ± 0.04 mbar (S2). The masks incorporating the S1 and S2 samples showed the highest resistance at this flow rate, with values of 0.80 ± 0.04 mbar and 0.81 ± 0.02 mbar, respectively. The masks with the S3 and S4 samples demonstrated intermediate resistance levels of 0.69 mbar and 0.68 ± 0.03 mbar. Notably, the mask with the S5 sample exhibited the lowest resistance at 0.61 ± 0.02 mbar, indicating potentially better breathability at lower airflow rates.

At the higher airflow rate of 95 L/min, the resistance values predictably increased for all mask samples. The range extended from 2.30 ± 0.10 mbar (S5) to 2.52 ± 0.13 mbar (S2). The trend observed at the lower flow rate was generally maintained, with the masks incorporating S1 and S2 showing the highest resistance (2.50 ± 0.13 mbar and 2.52 ± 0.13 mbar, respectively), S3 and S4 presenting intermediate values (2.44 ± 0.12 mbar and 2.42 ± 0.12 mbar), and S5 again demonstrating the lowest resistance at 2.30 ± 0.10 mbar.

The inhalation resistance measurements provide critical insights into the breathability and potential user comfort of the multi-layered masks incorporating different spun-bond nonwoven fabrics as their upper layer. Lower resistance values generally indicate better breathability, which is essential for user comfort and compliance of standards [35], especially during prolonged wear [44]. In this context, the mask sample incorporating the S5 sample stands out with the lowest resistance at both flow rates, suggesting that it may offer the best breathability among the tested masks.

The observed differences in inhalation resistance among the mask samples likely stem from variations in their overall structure, including the properties of the spun-bond nonwoven fabric upper layer and the interactions between different layers. The consistently lower resistance of the mask with the S5 sample aligns with the previously noted characteristics of its upper layer, including lower light permeability and higher tensile strength. This suggests that the S5 mask may have a more effective overall structure, allowing for easier air passage while maintaining protective properties. Conversely, the higher resistance values of masks with the S1 and S2 samples might indicate a denser or less permeable structure, which could offer better filtration but at the cost of an increased breathing effort.

It is important to note that, while lower breathing resistance is generally desirable for user comfort, it must be balanced against filtration efficiency. The ideal mask should offer a combination of adequate filtration and low breathing resistance. The resistance values observed here, particularly at the higher flow rate of 95 L/min, are all within a range that suggests acceptable breathability for mask use. However, the differences among the samples, though seemingly small, could become significant during extended wear periods. For instance, the difference of ~0.22 mbar between the S5 and S2 masks at 95 L/min could translate to noticeably reduced user fatigue over time. These findings underscore the importance of considering both inhalation resistance and filtration efficiency in the selection and design of multi-layered masks, with the mask incorporating the S5 sample potentially offering an advantageous balance of these properties.

Table 5 presents the average resistance to exhalation at a flow rate of 160 L/min for five multi-layered mask samples (S1–S5), each incorporating a different spun-bond nonwoven fabric as the upper layer. The measurements were taken in the following five different directions: forward; upward; downward; toward the left side; and toward the right side. This comprehensive approach allowed for a thorough assessment of the masks’ performance under various orientations, simulating real-world usage conditions.

Sample S5 consistently showed the lowest exhalation resistance across all directions, with values ranging from 2.38 ± 0.10 mbar (toward the right side) to 2.47 ± 0.10 mbar (upward). In contrast, sample S2 exhibited the highest resistance in most directions, with values ranging from 3.32 ± 0.17 mbar (toward the right side) to 3.57 ± 0.18 mbar (forward and downward). Samples S1, S3, and S4 demonstrated intermediate resistance levels, with samples S3 and S4 showing slightly lower resistance compared to sample S1. Notably, there were small variations in resistance depending on the direction of airflow for each mask sample, which could indicate slight anisotropic properties in the overall mask structure or variations in the layering of materials.

The directional variations in resistance were most pronounced in sample S2, with a difference of 0.25 mbar between its highest and lowest values. In contrast, sample S5 showed the lowest variation among the directions, with only a 0.09 mbar difference between its highest and lowest resistance values. This suggests that the mask incorporating the S5 sample may have a more uniform structure, providing consistent breathability regardless of orientation.

The exhalation resistance measurements at 160 L/min provide crucial insights into the performance of these multi-layered mask samples under high airflow conditions. The consistently lower resistance values exhibited by the mask incorporating the S5 sample across all directions indicate its potential as a superior design, offering improved breathability, even under demanding conditions. The variations in resistance across different directions for each mask sample highlight the importance of considering the overall mask structure and material layering in design. The more consistent performance of the mask with the S5 sample across all directions suggests a more uniform structure, potentially contributing to better user comfort regardless of mask positioning.

The higher resistance values observed in the mask with the S2 sample, particularly in the forward (3.57 ± 0.18 mbar) and downward (3.57 ± 0.18 mbar) directions, could lead to increased user discomfort and respiratory effort, especially during extended wear or physical activity. This could potentially result in reduced compliance with mask-wearing guidelines. Conversely, the lower and more consistent resistance of the mask with the S5 sample might encourage better adherence to mask-wearing protocols by minimizing user discomfort. However, it is crucial to balance lower exhalation resistance with effective particle filtration, as the ideal mask should provide adequate protection while minimizing breathing resistance.

It is important to evaluate these results in the context of the established standards for respiratory protective devices. The standard EN 149:2001+A1:2009 [35] stipulates maximum permitted resistances of 0.7 mbar for inhalation at 30 L/min, 2.4 mbar for inhalation at 95 L/min, and 3.0 mbar for exhalation at 160 L/min. Comparing our results to these standards, we can see that the mask incorporating the S5 sample consistently meets or exceeds these requirements across all test conditions. At 30 L/min inhalation, S5’s resistance of 0.61 ± 0.02 mbar is well below the 0.7 mbar limit. For 95 L/min inhalation, its 2.30 ± 0.10 mbar resistance is comfortably under the 2.4 mbar threshold. Finally, for exhalation at 160 L/min, S5’s highest recorded resistance of 2.47 ± 0.10 mbar is significantly below the 3.0 mbar maximum. This performance underscores the potential of the S5 mask to meet the FFP2 classification standards, highlighting its suitability for applications requiring this level of respiratory protection.

These findings underscore the complexity of mask design and highlight the need for a multifaceted approach to evaluation. Factors such as directional resistance, overall breathability, and potential anisotropic properties should be considered alongside filtration efficiency, durability, and other relevant parameters. The results from this study provide valuable data to inform the design process for more effective and comfortable protective masks, emphasizing the importance of both material selection and overall mask structure in achieving optimal performance.

### 3.6. Filter Penetration Test Results

Table 6 presents the results of two critical tests for the five multi-layered mask samples (with S1–S5), as follows: the paraffin oil penetration test and exposure to 120 mg of paraffin oil. These tests are essential for evaluating the filtration efficiency of the masks against oil-based particles, which can be particularly challenging to filter. For the paraffin oil penetration tests and exposure measurements presented in Table 6, it should be noted that the expanded uncertainty is ± 10% of the value of the measured result for a probability of coverage of 95%. This uncertainty applies to all penetration percentages reported in Table 6.

In the paraffin oil penetration test, the average penetration values ranged from 4.37 ± 0.15% (S5) to 8.80 ± 0.44% (S2). The mask incorporating S5 showed 4.37 ± 0.15%, the lowest penetration (compared to samples S1–S4), indicating the highest filtration efficiency against paraffin oil particles. The masks with the S3 and S4 samples demonstrated similar intermediate levels of penetration at 6.05 ± 0.30% and 6.01 ± 0.30%, respectively. The masks with the S1 and S2 samples showed the highest penetration values at 7.80 ± 0.39% and 8.80 ± 0.44%, suggesting lower filtration efficiency for oil-based particles.

The test involving exposure to 120 mg of paraffin oil, which simulates more prolonged exposure, showed similar trends. The maximum penetration values ranged from 4.28 ± 0.15% (S5) to 8.79 ± 0.44% (S2). Again, the mask with S5 performed best, showing the lowest maximum penetration. Interestingly, for most samples, the maximum penetration values were very close to the average values from the standard penetration test, indicating consistent performance, even under extended exposure.

The results of the paraffin oil penetration tests provide crucial insights into the filtration efficiency of these multi-layered masks against oil-based particles. The superior performance of the mask incorporating S5, with the lowest penetration values in both tests, suggests that its overall structure and material composition are particularly effective at trapping oil-based particles. This high filtration efficiency, combined with its previously observed low breathing resistance, indicates that the S5 mask may offer an optimal balance of protection and breathability.

The consistent performance of most masks between the standard penetration test and the 120 mg exposure test is noteworthy. This suggests that the masks maintained their filtration efficiency even under prolonged exposure to oil-based particles, which is crucial for real-world applications where masks may be worn for extended periods. However, the slightly higher penetration values for some samples (particularly S1 and S2) in the extended exposure test highlight the importance of considering long-term performance in mask design and material selection.

When evaluating these results, it is crucial to consider the established standards for respiratory protective devices. The standard EN 149:2001+A1:2009 [35] stipulates a maximum penetration of 6% at a flow rate of 95 L/min for the FFP2 classification. Comparing our results to this standard, we can see that the mask incorporating S5 clearly met and exceeded this requirement, with an average penetration of 4.37 ± 0.15% and a maximum penetration of 4.28 ± 0.15% under extended exposure. This performance is well below the 6% threshold, indicating filtration efficiency against oil-based particles. The masks with S3 and S4 samples also met the FFP2 requirement, albeit with a lower margin. However, the masks incorporating the S1 and S2 samples exceeded the 6% limit, suggesting they may not qualify for the FFP2 classification based on this criterion alone. These findings further emphasize the exceptional performance of the S5 mask, demonstrating its potential to meet and exceed FFP2 standards for protection against oil-based particulates.

The variation in penetration values across the different mask samples underscores the significant impact that material selection and the overall mask structure can have on filtration efficiency. While all tested masks showed penetration values below 9%, which may be acceptable depending on the specific application and regulatory standards, the difference between the best-performing (S5) and worst-performing (S2) masks is substantial. This difference could be critical in high-risk environments or for individuals with respiratory vulnerabilities. These findings emphasize the need for careful consideration of materials and design in mask production. Further research into the specific structural and material properties contributing to the performance of the S5 mask could inform the development of even more effective protective masks in the future.

In summary, our comprehensive analysis of five spun-bond nonwoven fabrics and their performance in multi-layered mask structures reveals significant variations in physical and mechanical properties. The 3D volumetric structure of samples S1–S4 contributes to higher light permeability but also results in an uneven fabric structure. In contrast, sample S5 consistently outperformed the others across multiple parameters. It demonstrated superior tensile strength, lower light permeability, and optimal performance in the breathability and filtration efficiency tests. Notably, the mask incorporating the S5 sample met or exceeded the FFP2 standards for both breathing resistance and paraffin oil penetration. These findings highlight the critical impact of material selection and structural design on mask performance. The described characteristics of S5 suggest that a balance between the material density, uniformity, and overall mask structure is key to achieving high-performance protective masks that offer both effective filtration and user comfort.

## 4. Conclusions

Based on the comprehensive analysis of the five spun-bond nonwoven fabric samples (S1–S5) used in multi-layered mask construction, the following conclusions can be drawn.

Our study demonstrates that the structural properties of spun-bond nonwoven fabrics significantly impact the performance of multi-layered protective masks. The S5 material consistently exhibited superior breathability and filtration efficiency, meeting or exceeding the EN 149:2001+A1:2009 standards. Notably, S5 showed a 45% lower average penetration of paraffin oil compared to the next best-performing fabric. These findings highlight the critical role of material selection and structural design in mask production, particularly for high-protection applications. Future research should focus on optimizing material properties and mask structure, using S5 as a benchmark, to enhance both protective capabilities and user comfort. Our results provide valuable insights for manufacturers and healthcare professionals in selecting materials for effective PPE design.

## Figures and Tables

**Table 1:** Light permeability of spun-bond nonwoven fabrics.

No.	Reference Number	Area of Light Passed (%)
1	S1	14.84 ± 0.70
2	S2	14.44 ± 0.68
3	S3	15.80 ± 0.84
4	S4	15.05 ± 0.70
5	S5	10.09 ± 0.50

**Table 2:** Mass determination per unit area of five types of specimens.

No.	Reference Number	Mass, g/m^2^	Thickness (mm)
1	S1	53 ± 2	0.29 ± 0.03
2	S2	50 ± 2	0.25 ± 0.02
3	S3	51 ± 2	0.25 ± 0.02
4	S4	48 ± 2	0.24 ± 0.02
5	S5	50 ± 2	0.21 ± 0.02

**Table 3:** Average elongation and tensile strength of five types of specimens.

No.	Reference Number	Ultimate Tensile Strength (MPa)		Elongation (%)	
		MD	CMD	MD	CMD
1	S1	5.4 ± 0.4	3.4 ± 0.3	208 ± 18	172 ± 14
2	S2	6.7 ± 0.5	3.9 ± 0.3	218 ± 20	184 ± 16
3	S3	5.7 ± 0.5	3.6 ± 0.3	223 ± 19	210 ± 19
4	S4	6.0 ± 0.5	4.2 ± 0.4	239 ± 19	241 ± 21
5	S5	13.5 ± 0.6	5.6 ± 0.3	135 ± 7	153 ± 8

**Table 4:** Average resistance to inhalations (30 and 95 L/min).

No.	Reference Number	Average Resistance to Inhalation at 30 L/min (mbar)	Average Resistance to Inhalation at 95 L/min (mbar)
1	S1	0.80 ± 0.04	2.50 ± 0.13
2	S2	0.81 ± 0.04	2.52 ± 0.13
3	S3	0.69 ± 0.03	2.44 ± 0.12
4	S4	0.68 ± 0.03	2.42 ± 0.12
5	S5	0.61 ± 0.02	2.30 ± 0.10

**Table 5:** Average resistance to inhalations (160 L/min).

No.	Reference Number	Forward (mbar)	Upward (mbar)	Downward (mbar)	Toward the Left Side (mbar)	Toward the Right Side (mbar)
1	S1	3.18 ± 0.16	3.21 ± 0.16	3.22 ± 0.16	3.22 ± 0.16	3.26 ± 0.16
2	S2	3.57 ± 0.18	3.56 ± 0.18	3.57 ± 0.18	3.48 ± 0.17	3.32 ± 0.17
3	S3	2.98 ± 0.15	2.99 ± 0.15	3.00 ± 0.15	2.9 ± 0.15	2.89 ± 0.14
4	S4	2.99 ± 0.15	2.98 ± 0.15	3.00 ± 0.15	3.01 ± 0.15	3.00 ± 0.15
5	S5	2.46 ± 0.10	2.47 ± 0.10	2.45 ± 0.10	2.41 ± 0.10	2.38 ± 0.10

**Table 6:** Paraffin oil penetration test and exposure to 120 mg of paraffin oil.

No.	Reference Number	Paraffin Oil Penetration Test	Exposure to 120 mg of Paraffin Oil
		Average Value of Penetration %	Max. Value of Penetration %
1	S1	7.80 ± 0.39	8.02 ± 0.40
2	S2	8.80 ± 0.44	8.79 ± 0.44
3	S3	6.05 ± 0.30	5.99 ± 0.30
4	S4	6.01 ± 0.30	6.02 ± 0.30
5	S5	4.37 ± 0.15	4.28 ± 0.15

## Data Availability

The original contributions presented in the study are included in the article, further inquiries can be directed to the corresponding authors.
